# Bibliometric analysis of the intestinal microbiota and demyelinating diseases, particularly multiple sclerosis, since 2014

**DOI:** 10.3389/fnins.2025.1506566

**Published:** 2025-03-05

**Authors:** Ling Chen, Le-Le Wu, Chang-Yin Yu, Zu-Cai Xu, Hao Huang

**Affiliations:** ^1^Department of Neurology, Affiliated Hospital of Zunyi Medical University, Zunyi, China; ^2^Department of Neurology, Xinqiao Hospital and the Second Affiliated Hospital, Army Medical University (Third Military Medical University), Chongqing, China

**Keywords:** intestinal microbiota, multiple sclerosis, demyelinating diseases, bibliometrics, visualization analysis

## Abstract

**Background:**

The gut–brain axis (GBA) represents a complex, bidirectional communication network that connects the central nervous system (CNS) and the gastrointestinal system. Our study aimed to explore the correlation between the intestinal microbiota and demyelinating diseases from a bibliometric perspective, focusing on research since 2014.

**Methods:**

A comprehensive search was carried out on the Web of Science Core Collection (WoSCC) to locate studies on the intestinal microbiota and demyelinating diseases, with a focus on publications from 1 January 2014 to 29 March 2024. We visualized and analyzed the data using VOSviewer, CiteSpace, and Charticulator.

**Results:**

We gathered 429 scholarly articles on the intestinal microbiota and demyelinating disorders published in the past 10 years. Research concerning the intestinal microbiota and demyelinating diseases has demonstrated a consistent increase in frequency over time. The USA has the highest number of publications, while Canada has the highest average number of citations, reaching as high as 3,429, which is greater than that of the USA. Moreover, the journal with the highest number of publications was Frontiers in Immunology, with 33 publications and 1,494 citations. The majority of the scholars focused on “multiple sclerosis” and “gut microbiota,” which are the primary keywords in the field of the intestinal microbiota and demyelinating diseases.

**Conclusion:**

This study conducted a comprehensive analysis of existing research investigating the correlation between the intestinal microbiota and demyelinating diseases. Using advanced bibliometric tools such as VOSviewer and CiteSpace, this study analyzed the intricate relationship between the intestinal microbiota and the pathogenesis of demyelinating conditions. In addition, the study used literature statistical analysis to identify research hotspots and future directions in the field.

## 1 Introduction

A significant part of human physiology is the gut microbiota, which consists of a dynamic and intricate community of bacteria, fungi, viruses, and the metabolites they produce. It is responsible for regulating a wide range of vital processes necessary for maintaining homeostasis in the host, including metabolic, endocrine, nutritional, immune, and neurological functions (Cabrera-Mulero et al., 2019). Alterations in the gut microbiota can affect bodily homeostasis; conversely, shifts in body homeostasis can influence the gut microbiota (Wijesekara et al., 2024; Clemente et al., 2012). In other words, the relationship between the gut microbiota and human body function is bidirectional, functioning as a two-way communication system between the host and microbiome (Wang et al., 2023). Despite the anatomical separation between the gut and brain, the connection between the gut and brain through the nervous and immune systems provides multiple potential pathways for communication (Cryan et al., 2019). Over the past 20 years, the field of biomedicine has undergone a shift with the recognition that the gut microbiota and the microbiome play crucial roles in modulating the central nervous system (CNS). This recognition has opened new avenues for understanding the intricate relationships between gut microorganisms and brain function, marking the beginning of a new era in biomedical research (Cryan et al., 2020). In recent years, interest in the role and significance of bidirectional communication mediated by the gut–brain axis (GBA) in the etiology of diverse central nervous disorders has increased (Chidambaram et al., 2022; Wang and Kasper, 2014). This area of study, often characterized by intricate interactions between the brain and the gastrointestinal system, is rapidly emerging as one of the most promising and dynamic fields of research (Seguella et al., 2019; Heiss and Olofsson, 2019). A previous study revealed that the GBA contributes to normal CNS function and pathology (Cryan et al., 2019; Socała et al., 2021). For the past few years, a growing body of evidence has implicated dysregulation of the GBA in the pathophysiology of several neurological disorders, including Alzheimer’s disease (Dissanayaka et al., 2024), autism spectrum disorder (Kang et al., 2019), multiple sclerosis (MS) (Parodi and Kerlero de Rosbo, 2021), Parkinson’s disease, and stroke (Zhao et al., 2021; Bogiatzi et al., 2018). Some studies have demonstrated that fundamental neural processes, including development, myelination, neurogenesis, and microglial activation, are critically influenced by the composition of the microbiota (Gao et al., 2019; Luczynski et al., 2016). These findings further underscore the significant impact of microbial communities on the structural and functional maturation of the nervous system.

The myelin sheath encases the majority of nerve fibers in the central and peripheral nervous systems, and its primary objective is to increase nerve conduction and preserve the energy expended during the propagation of action potentials (Bernardo and Visentin, 2023). Demyelinating diseases are a group of disorders characterized by changes in myelin. MS is a chronic autoimmune inflammatory disorder of the CNS. This condition is characterized by the development of inflammatory lesions within the CNS, resulting in the degradation of myelin sheaths and subsequent damage to demyelinated axons (Thirion et al., 2023). The development of various pathological conditions is increasingly linked to the composition of the microbiota and the GBA. A previous study suggested that the GBA may be important in the onset and progression of MS (Preiningerova et al., 2022). Active lesions in MS exhibit inflammatory alterations, indicative of concurrent assault by autoreactive T and B lymphocytes in the white matter of the brain. The initiation of this autoimmune response has often been linked to environmental influences, with microbial infection standing out as a primary factor (Berer et al., 2011). In addition, Banati et al. reported that antibodies against gastrointestinal antigens may reflect alterations in the microbiota and immune responses in the gut. They found that patients with MS and neuromyelitis optica (NMO) had higher levels of antibodies against gastrointestinal antigens compared to healthy controls, indicating a close relationship between the intestinal microbiota and the immune response in patients with demyelinating diseases (Cheng et al., 2022). Studies have shown that alterations in the gut microbiota or its metabolites can modulate inflammation and demyelination, especially the intermittent fasting-induced enrichment of lactobacilli (Berer et al., 2011; Lee et al., 2011), which can reduce inflammatory immune responses (Umbrello and Esposito, 2016).

To date, significant controversy surrounds the extent and exact mechanisms through which the altered GBA may influence demyelination (Ghezzi et al., 2021). To address this knowledge gap, we conducted a bibliometric analysis using tools such as VOSviewer and CiteSpace to examine research trends and emerging frontiers in the fields of the intestinal microbiota and demyelinating diseases. This analysis focused on authors, key publications, journals, institutions, countries, and the research network of publications. Through this approach, we aimed to identify pivotal themes that characterize the intersection of these two critical areas of study. We identified Helen Tremlett as the most influential author, known for her work on the etiology, risk factors, and epidemiology of MS and gut microbiome research in demyelinating diseases. Moreover, we also found that the relationship between the intestinal microbiota and demyelinating diseases has garnered increasing attention by analyzing the annual publication volume of related literature. In general, the relationships and underlying processes linking the intestinal microbiota to demyelinating diseases remain prominent research topics.

## 2 Materials and methods

### 2.1 Method and data sources

Web of Science (WOS) is one of the most commonly used academic database sources (Wu et al., 2021). Data for the present study were retrieved from the Web of Science Core Collection (WOSCC) database starting 1 January 2014. To minimize bias stemming from daily updates in the database, we conducted the literature search on WOSCC in a single day on 29 March 2024. We visualized the comprehensive data using VOSviewer, CiteSpace, and Charticulator.

### 2.2 Retrieval strategies

**Figure 1:**
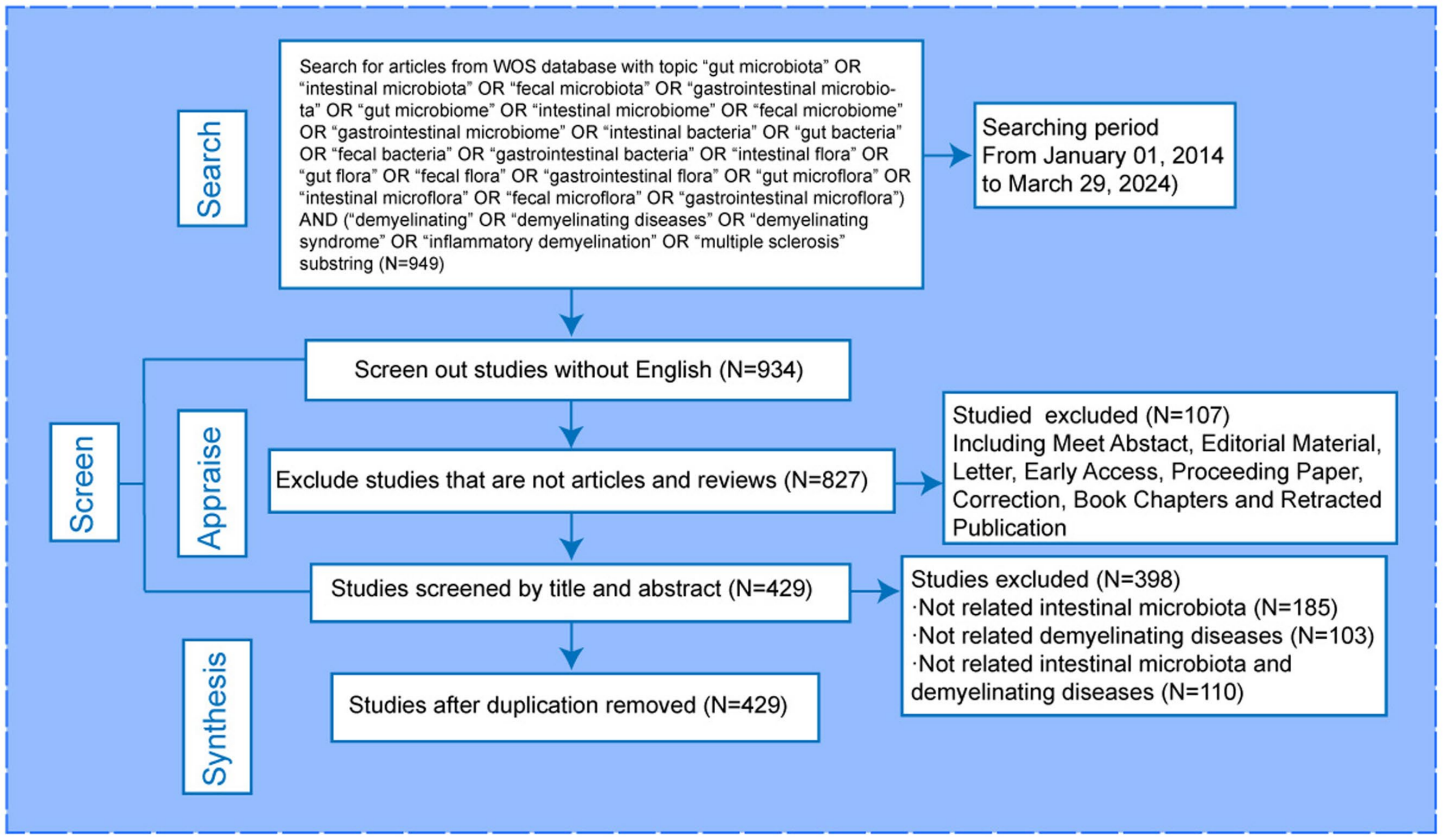
The selection of documents.

We used TS = ((“gut microbiota” OR “intestinal microbiota” OR “fecal microbiota” OR “gastrointestinal microbiota” OR “gut microbiome” OR “intestinal microbiome” OR “fecal microbiome” OR “gastrointestinal microbiome” OR “intestinal bacteria” OR “gut bacteria” OR “fecal bacteria” OR “gastrointestinal bacteria” OR “intestinal flora” OR “gut flora” OR “fecal flora” OR “gastrointestinal flora” OR “gut microflora” OR “intestinal microflora” OR “fecal microflora” OR “gastrointestinal microflora”) AND (“demyelinating” OR “demyelinating diseases” OR “demyelinating syndrome” OR “inflammatory demyelination” OR “multiple sclerosis”). The publication period was 1 January 2014, and original articles and reviews were the types of documents that were considered. A total of 949 publications were included, with 379 original articles and 448 reviews, all in English. The article selection process was conducted independently by Chen and Wu, with any disagreements resolved by a third researcher. We excluded studies that were not related to intestinal microbiota or demyelinating diseases. Figure 1 provides visual representations of the search strategy.

### 2.3 Data analysis and visualization

The software programs CiteSpace (version 6.1. R3) and VOSviewer (version 1.6.18) are crucial for performing data analysis and visualization and identifying hotspots and trends within scientific publications. In this study, we exported the records retrieved from WOS in plain text format. This file, which was given the name download_XX.txt, contained both complete records and references. Afterward, VOSviewer and CiteSpace were used for bibliometric and visual analysis of the imported data. Comprehensive analyses across a wide range of dimensions, including countries, journals, authors, research fronts, and emerging trends, were expertly performed using VOSviewer and CiteSpace. They were used to analyze the number of annual outputs, growth trends, and frequently used keywords, as well as to identify strong citation burst keywords over time. We used Charticulator to draw a chord diagram illustrating the relationships between countries.

## 3 Results

### 3.1 Annual growth trend of publication outputs

**Figure 2:**
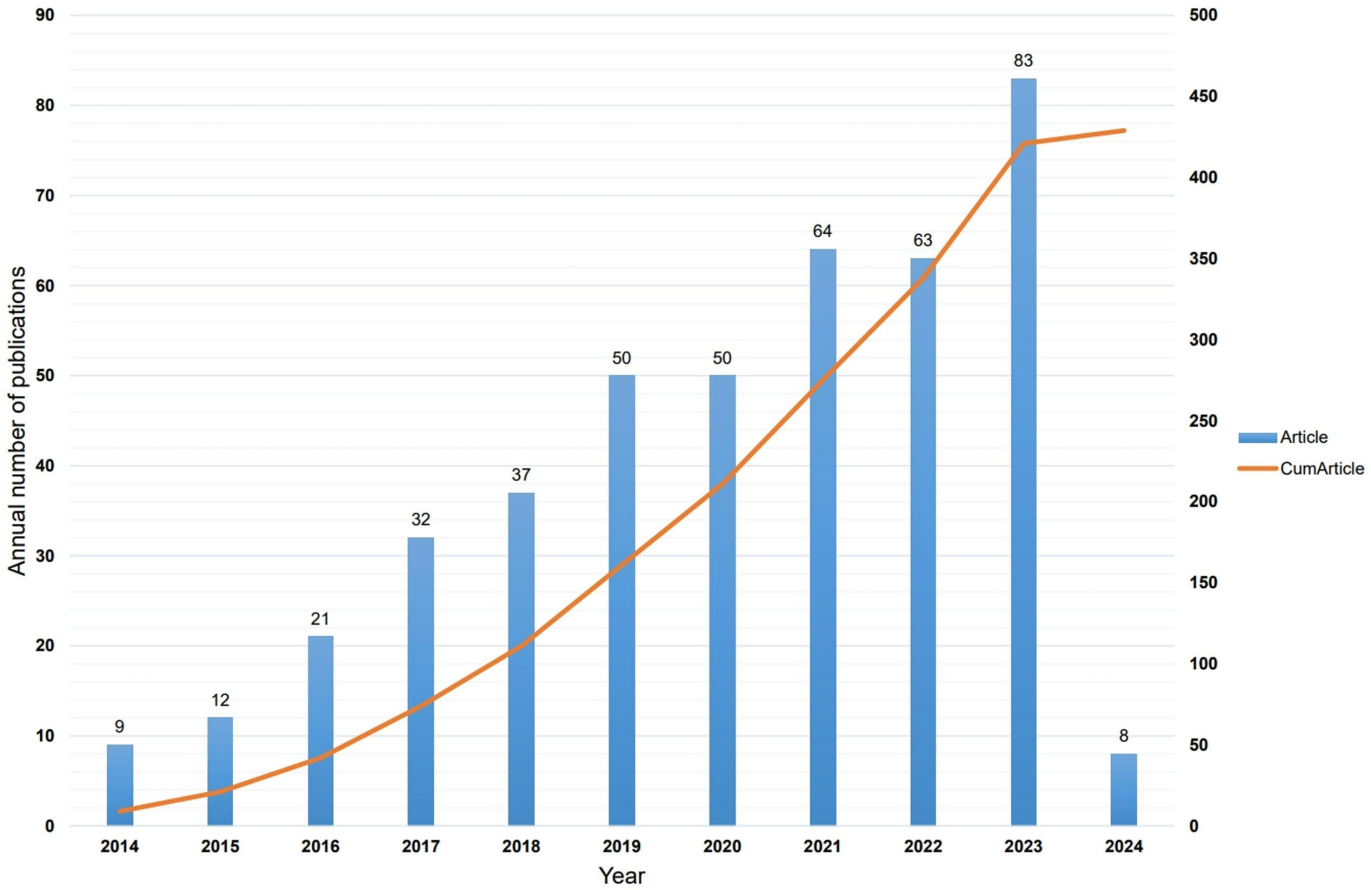
Distribution of publication on intestinal microbiota and demyelinating diseases since 2014.

A total of 429 articles were included in this analysis, authored by 2,397 individuals representing 821 institutions across 47 countries. The articles were published in 193 journals, citing 23,774 sources from 3,498 journals. Since 2014, 422 articles related to the intestinal microbiota and demyelinating diseases have been published, indicating an increasing trend with occasional oscillations (Figure 2). Overall, since 2014, the number of published articles on the intestinal microbiota and demyelinating diseases has shown an increasing trend. These findings suggest that, with advancements and progress in medicine, an increasing number of researchers are focusing on exploring the relationship between the intestinal microbiota and demyelinating diseases.

### 3.2 Authorship

**Figure 3:**
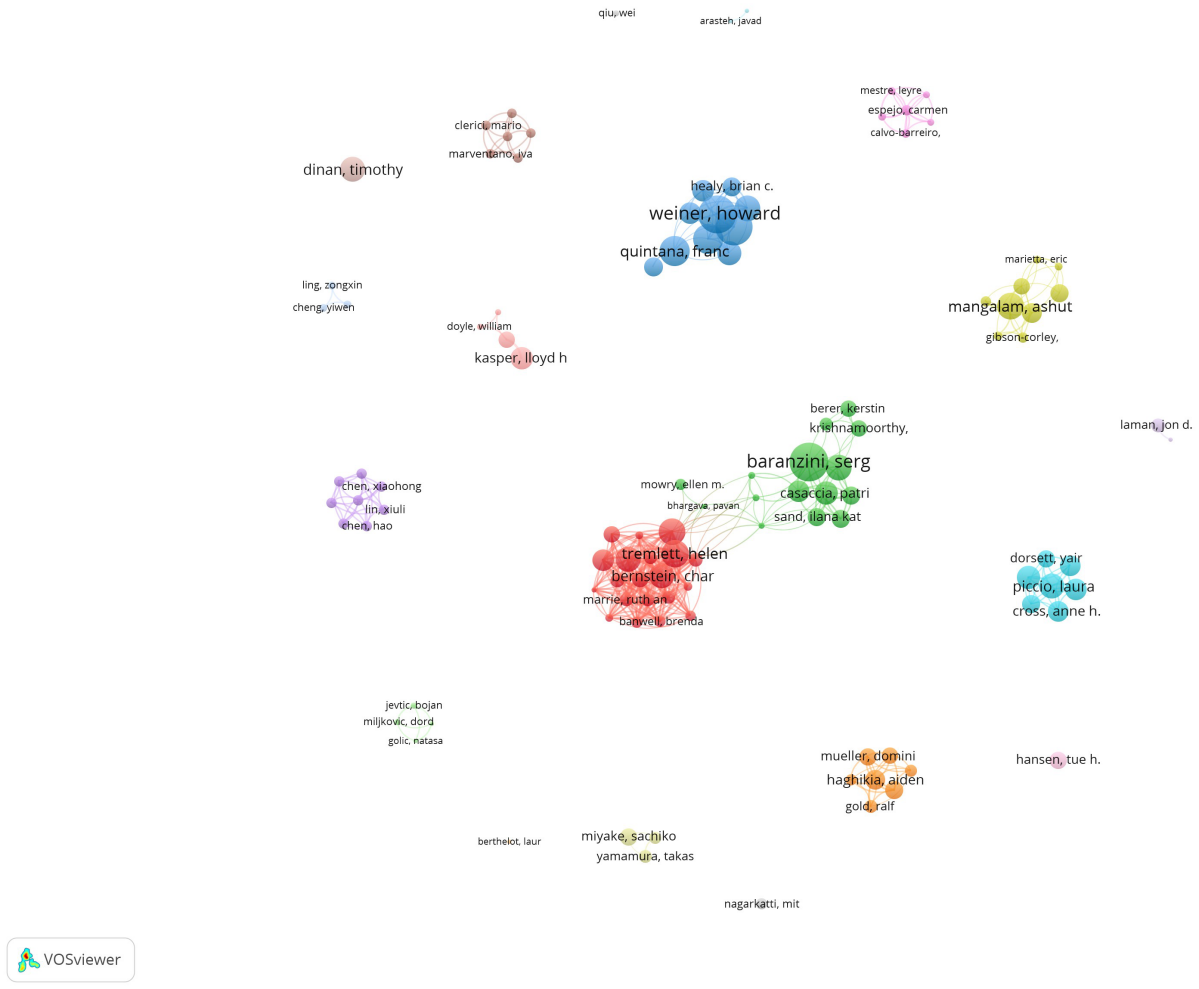
Network visualization of core authors. The larger the node, the greater the number of published articles. The node connection lines represent the strength of the relationship between authors.

The bibliometric principle known as Prices’ Law states that a core author in a given field must have produced a certain minimum number of articles: m = 0.749× $\sqrt{\text{nmax}\;}$ (n_max_ is the number of articles written by the most prolific authors in the field). In this study, m = 0.749× $\sqrt{14}$ ≈ 2.80. Thus, authors who have published three or more articles are considered core contributors to the field of the intestinal microbiota and demyelinating diseases. Since 2014, a total of 2,397 authors have contributed to the field of the intestinal microbiota and demyelinating diseases, and 111 authors are considered core authors, having published more than three articles (Figure 3).

The authors who have published more than 11 articles in this field are listed in Table 1, which displays the highly productive authors. The three authors with the most publications were found to be Helen Tremlett (14, 3.2%), Emmanuelle Waubant (13, 3.0%), and Lloyd H. Kasper (13, 3.0%). The most productive author was Helen Tremlett, having published 14 articles in this field. Her research has focused on the etiology, risk factors, and epidemiology of MS and gut microbiome research in demyelinating diseases. However, Lloyd H. Kasper was found to be the most influential author, having published 13 articles with the highest number of citations, with an average of 86.69 citations.

**Table 1:** Top five productive authors in the intestinal microbiota and demyelinating disease research field since 2014.

Rank	Author	Publications	Citations	Average citation
1	Helen Tremlett	14	659	47.07
2	Emmanuelle Waubant	13	857	65.92
3	Lloyd H. Kasper	13	1,127	86.69
4	Javier Ocho-reparaz	11	452	41.09
5	Ashutosh K. Mangalam	11	854	77.64

### 3.3 Countries and organizations

**Figure 4:**
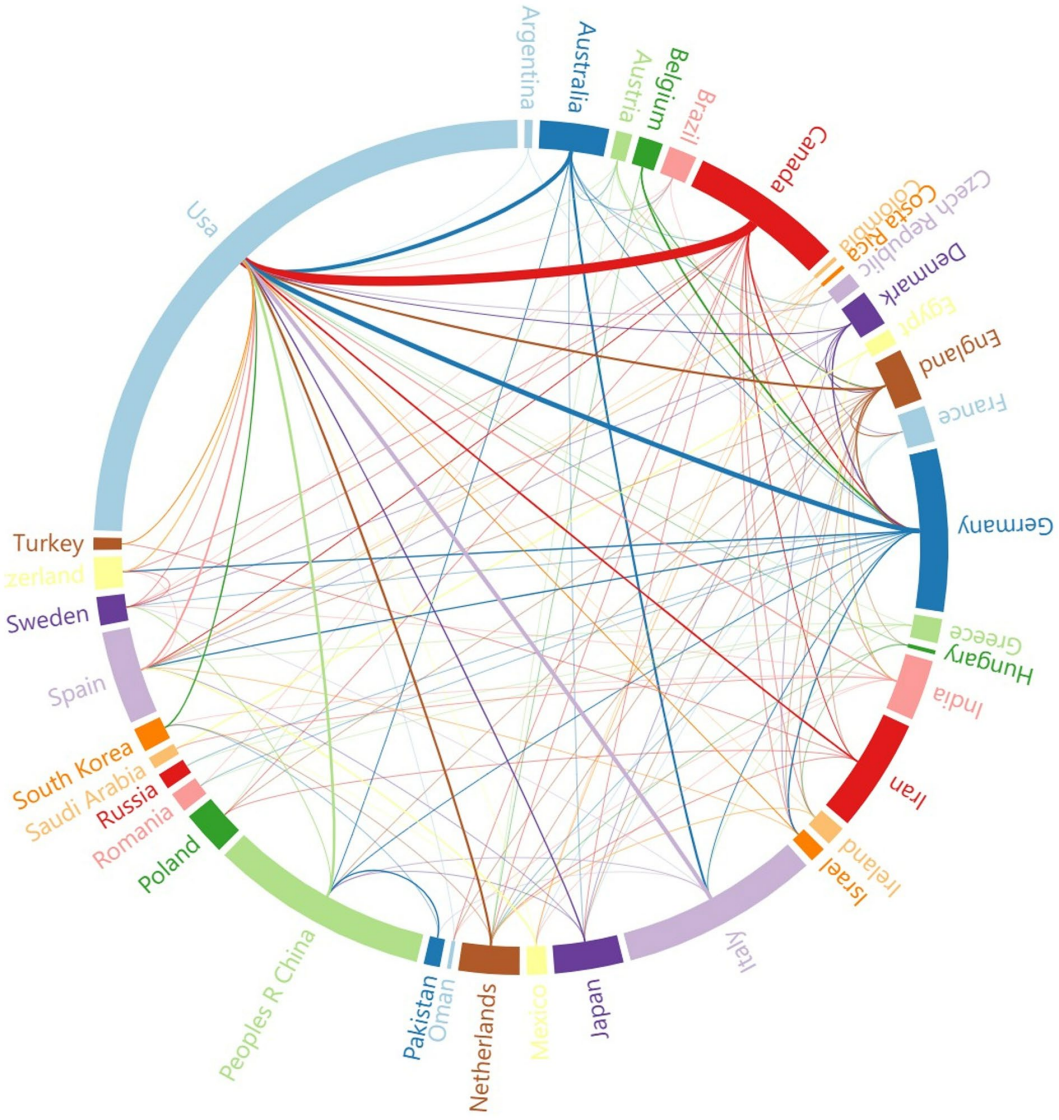
The cooperative relationship between countries. The thickness of the line represents the strength of the collaboration between countries.

Since 2014, 47 countries have published articles in the field of the intestinal microbiota and demyelinating diseases. Figure 4 shows the cooperative relationships between countries via VOSviewer and Microsoft Charticulator, and Table 2 presents the top five countries with the highest number of articles published, along with their respective publication and citation counts. To further analyze the high-productivity countries, we found that the United States has published the most articles (158, 36.8%), followed by the People’s Republic of China (54, 12.6%) and Italy (49, 11.4%). Notably, Canada has only 38 publications, but the number of citations is as high as 3,429, which is greater than that of the USA, the People’s Republic of China, and Italy and is closely related to Helen Tremlett. Helen Tremlett has contributed to the study of demyelinating diseases with numerous publications in high-impact journals. Furthermore, Microsoft Charticulator revealed that the most frequent collaboration has been between the United States and Canada (Figure 4).

**Table 2:** Top five countries and organizations for publications in the field of the intestinal microbiota and demyelinating diseases.

Rank	Country	Publications	Citations	Organization	Publications	Citations
1	USA	158	12,488	The University of California, San Francisco	28	2,771
2	The People’s Republic of China	54	1,760	The University of British Columbia	19	1,212
3	Italy	49	2,682	Harvard Medical School	14	1,591
4	Germany	40	3,567	Mayo Clinic	12	1,109
5	Canada	38	3,429	The University of Iowa	12	1,038

**Figure 5:**
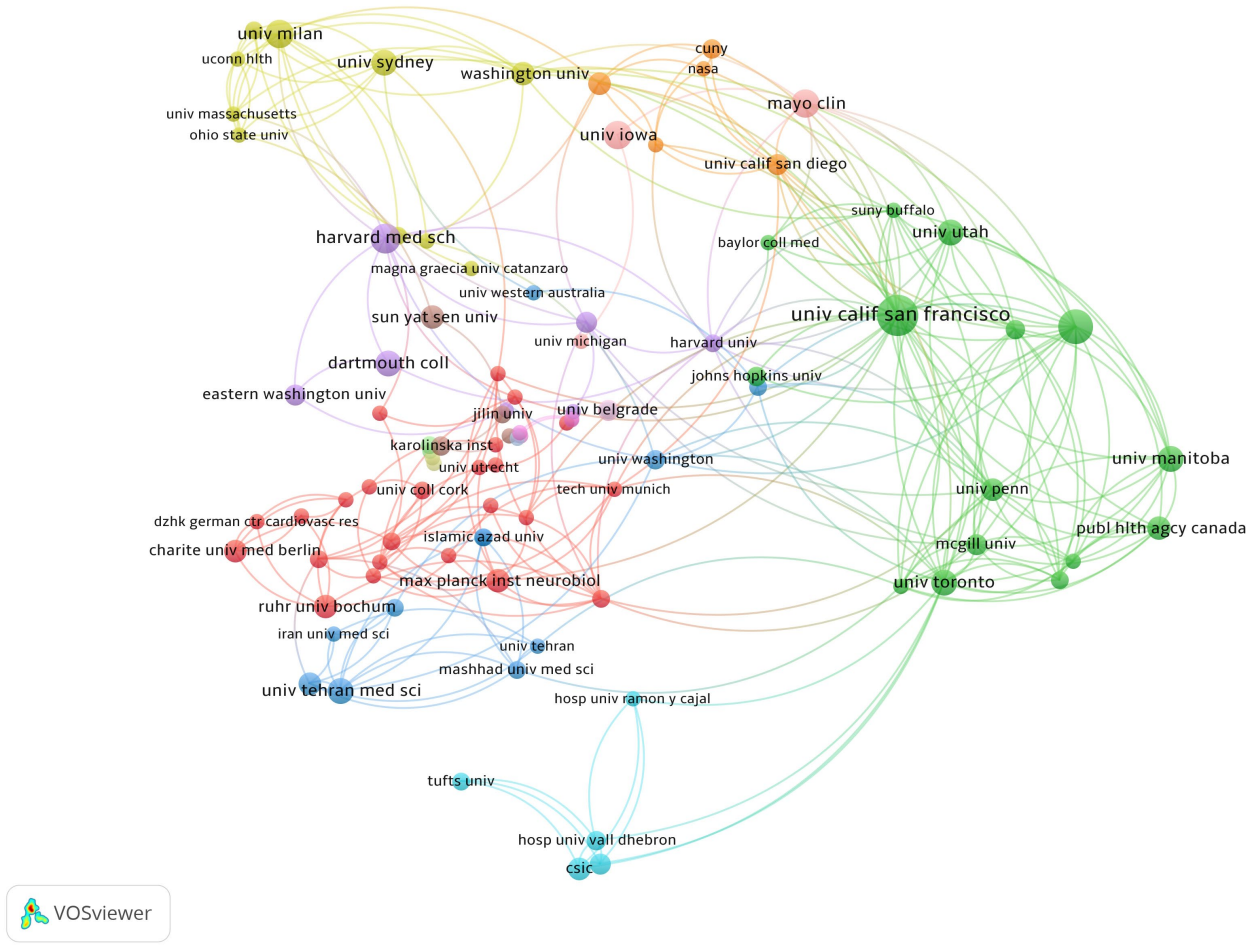
Cooperation map of intestinal microbiota and demyelinating diseases. The larger the node, the greater the publications, and the node connection lines represent the strength of the relationship between organizations.

An examination of institutional collaboration was carried out using VOSviewer, which ultimately led to the creation of a network map of institutional collaboration (Figure 5). The minimum publication threshold was set at 3, and 101 institutions met this criteria. As depicted in Figure 5, these institutions demonstrated close collaboration. The most significant research institution was found to be the University of California, San Francisco, which has the highest number of publications and citations. The University of British Columbia ranked second with 19 publications and 1,212 total citations. Among the top five organizations, four were from the USA and one was from Canada (Table 2). Moreover, among the top three authors in terms of publications, Helen Tremlett is affiliated with the University of British Columbia, followed by Emmanuelle Waubant and Lloyd H. Kasper, both of whom are from the USA, with Emmanuelle Waubant from the University of California, San Francisco. This observation is consistent with our previous research on authorship, further substantiating the reliability of our study.

### 3.4 Journals

**Figure 6:**
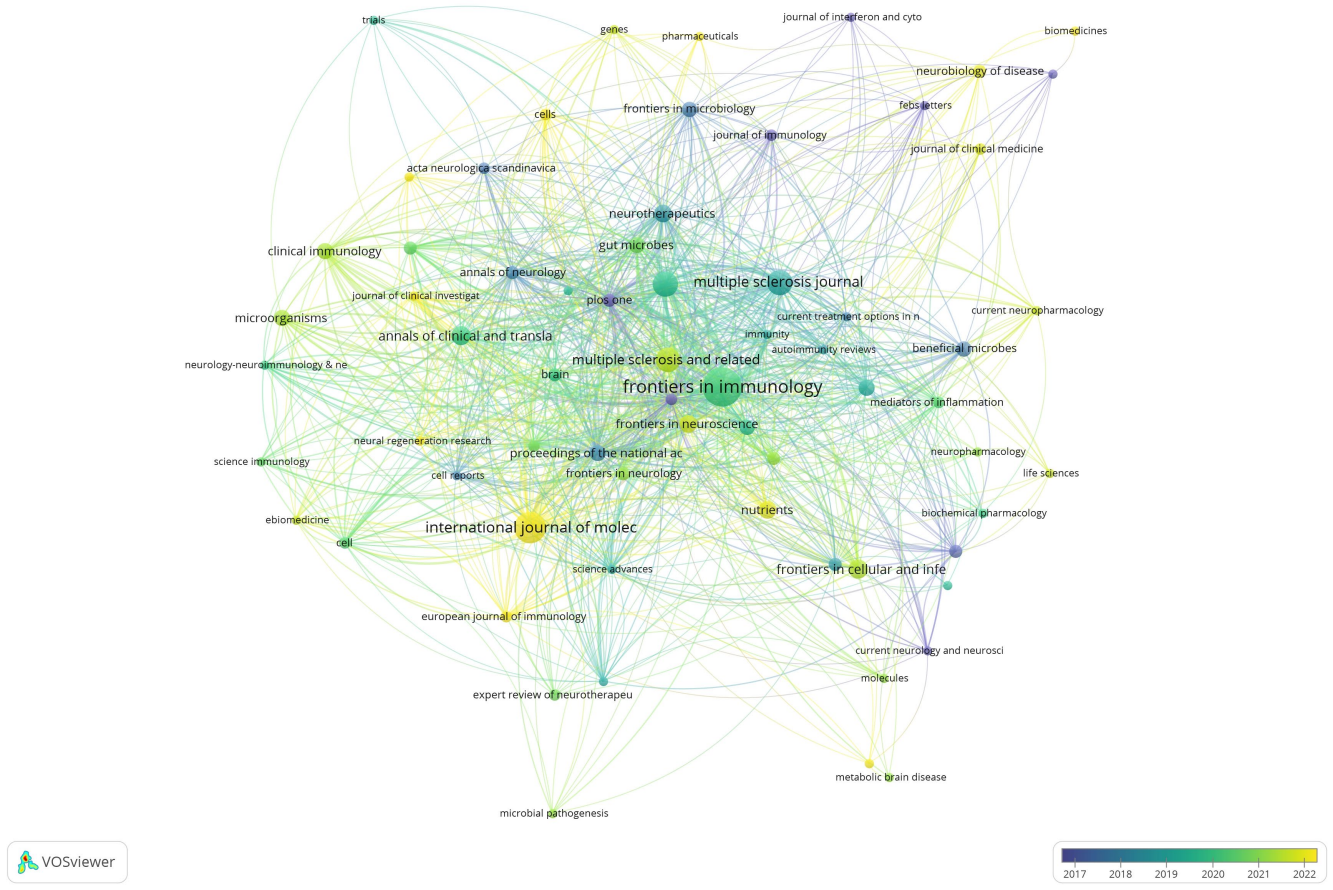
Visualization of the journal network. The larger the node is, the greater the volume of published articles and the node connection lines represent the strength of the relationship between journals. The darker the color, the earlier the article was published, and the lighter the color, the later the article was published.

A total of 429 articles related to the intestinal microbiota and demyelinating diseases have been published across 193 journals. Figure 6 shows that after 2019, the number of articles published in the majority of the journals reached its highest point. The field of the intestinal microbiota and demyelinating diseases has developed rapidly in the past 5 years, which is consistent with the findings shown in Figure 2. Table 3 lists the journals with the most publications in the top 10. Frontiers in Immunology was found to be the journal with the highest number of publications, with 33 articles, accounting for 7.7%. The International Journal of Molecular Sciences came in second with 21 articles, accounting for 4.9%, followed by Scientific Reports and the Multiple Sclerosis Journal with 14 articles, accounting for 3.3%. The journal with the most average citations was Scientific Reports, which published just 14 articles.

**Table 3:** Top 10 journals: the number of publications in intestinal microbiota and demyelinating disease research.

Rank	Source	Publication	Citations	Average Citation/Publication
1	Frontiers in Immunology	33	1,494	45.27
2	International Journal of Molecular Sciences	21	300	14.29
3	Scientific Reports	14	881	62.93
4	Multiple Sclerosis Journal	14	428	30.57
5	Multiple Sclerosis and Related Disorders	13	232	17.85
6	Frontiers in Cellular and Infection Microbiology	8	277	34.63
7	Annals of Clinical and Translational Neurology	8	175	21.88
8	Nutrients	7	140	20.00
9	Neurotherapeutics	7	429	61.29
10	Frontiers in Neuroscience	7	276	39.43

### 3.5 Frequency and citation bursts of keywords

**Figure 7:**
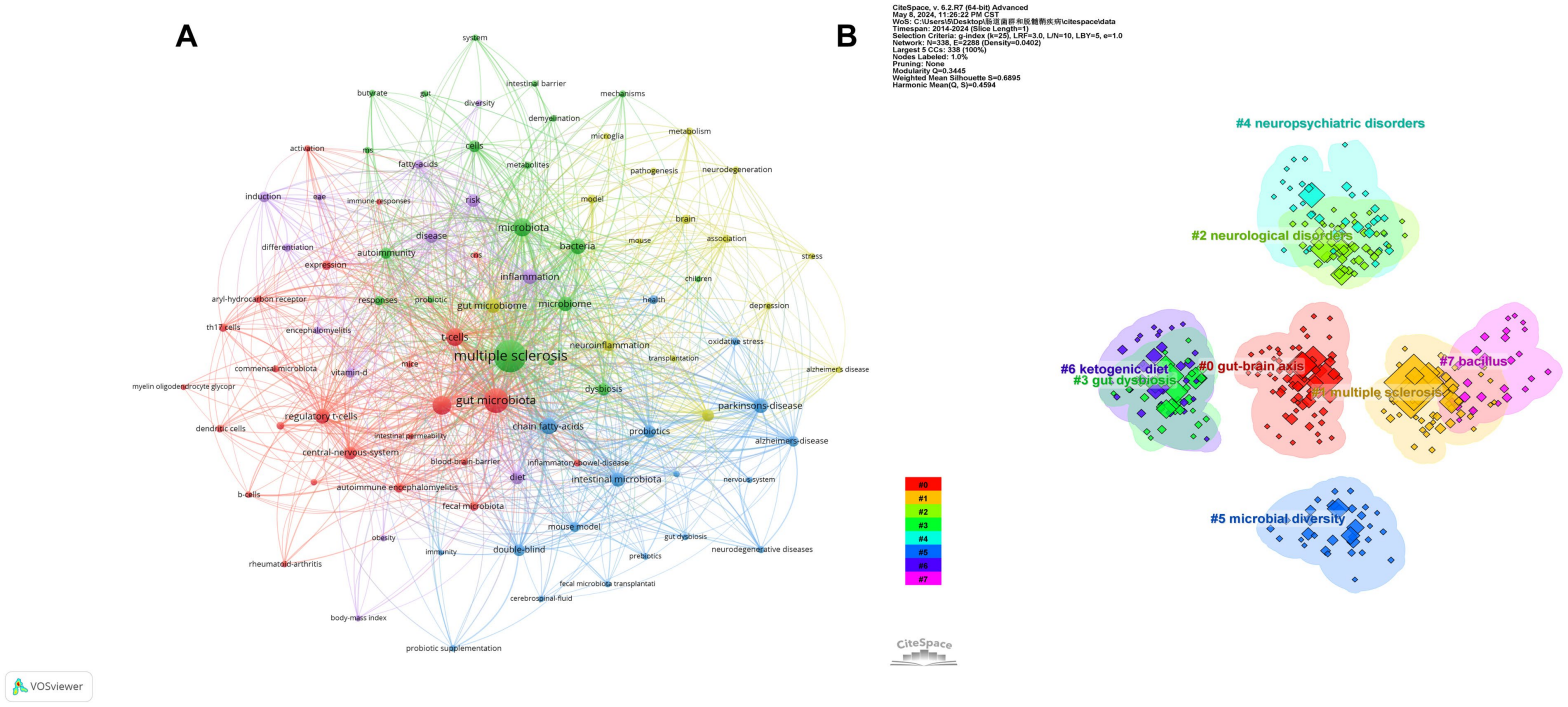
**(A)** The network map and cluster of keywords in intestinal microbiota and demyelinating diseases. The larger the node, the greater the frequency of keywords that appear. The node connection lines represent the strength of the relationship between keywords. **(B)** Visualization of the keywords cluster analysis. Each cluster is depicted with a unique color.

Keyword frequency reveals the focus and hot spots of a research field. Thus our analysis was conducted on 1,785 keywords in the intestinal microbiota and demyelinating disease research field using VOSviewer software (Figure 7A). We analyzed the frequency of co-occurring keywords related to the intestinal microbiota and demyelinating diseases. The larger the node

the higher the frequency of keyword co-occurrence. The common top 10 keywords are listed in Table 4. The top three keywords were multiple sclerosis (frequency: 283), gut microbiota (frequency: 166), and experimental autoimmune encephalomyelitis (frequency: 94).

**Table 4:** Frequency of co-occurring keywords in the top 10.

Rank	Keyword	Frequency	Cluster-ID	Silhouette	Mean (Year)	Included keywords (Top three)
1	Multiple sclerosis	284	#0	0.604	2016	Gut–brain axis; dendritic cells; regulatory t cells;
2	Gut microbiota	166	#1	0.729	2016	multiple sclerosis; 16 s RNA; gut microbiota;
3	Experimental autoimmune encephalomyelitis	94	#2	0.645	2018	neurological disorders; neurodegenerative diseases; alpha-synuclein;
4	Microbiota	88	#3	0.721	2017	gut dysbiosis; Vitamin D; gut microbiome;
5	T-cell	82	#4	0.747	2019	neuropsychiatric disorders; microbiota–gut–brain axis; intestinal microbiota;
6	Chain fatty acids	67	#5	0.74	2020	microbial diversity; type 1 diabetes; systemic lupus erythematosus;
7	Bacteria	65	#6	0.662	2019	ketogenic diet; TGF-beta; epilepsy;
8	Inflammation	60	#7	0.811	2021	bacillus; fermented camels milk; postbiotics;
9	Parkinson’s-disease	59	N/A	N/A	N/A	N/A
10	Regulatory T-cell	57	N/A	N/A	N/A	N/A

The keywords related to the intestinal microbiota and demyelinating diseases were grouped into eight clusters using CiteSpace. These clusters were as follows: #0 for the gut–brain axis, #1 for multiple sclerosis, #2 for neurological disorders, #3 for gut dysbiosis, #4 for neuropsychiatric disorders, #5 for microbial diversity, #6 for the ketogenic diet, and #7 for bacillus (Figure 7B). A cluster’s average silhouette (S-value) is used as an indicator to evaluate clustering. According to earlier research, a silhouette coefficient (S) exceeding 0.5 is deemed acceptable, whereas an S-value greater than 0.7 signifies strong clustering evidence (Wang et al., 2022). In our study, each of the obtained clusters had an S-value greater than 0.5, suggesting that our results are reasonable (Table 4).

**Figure 8:**
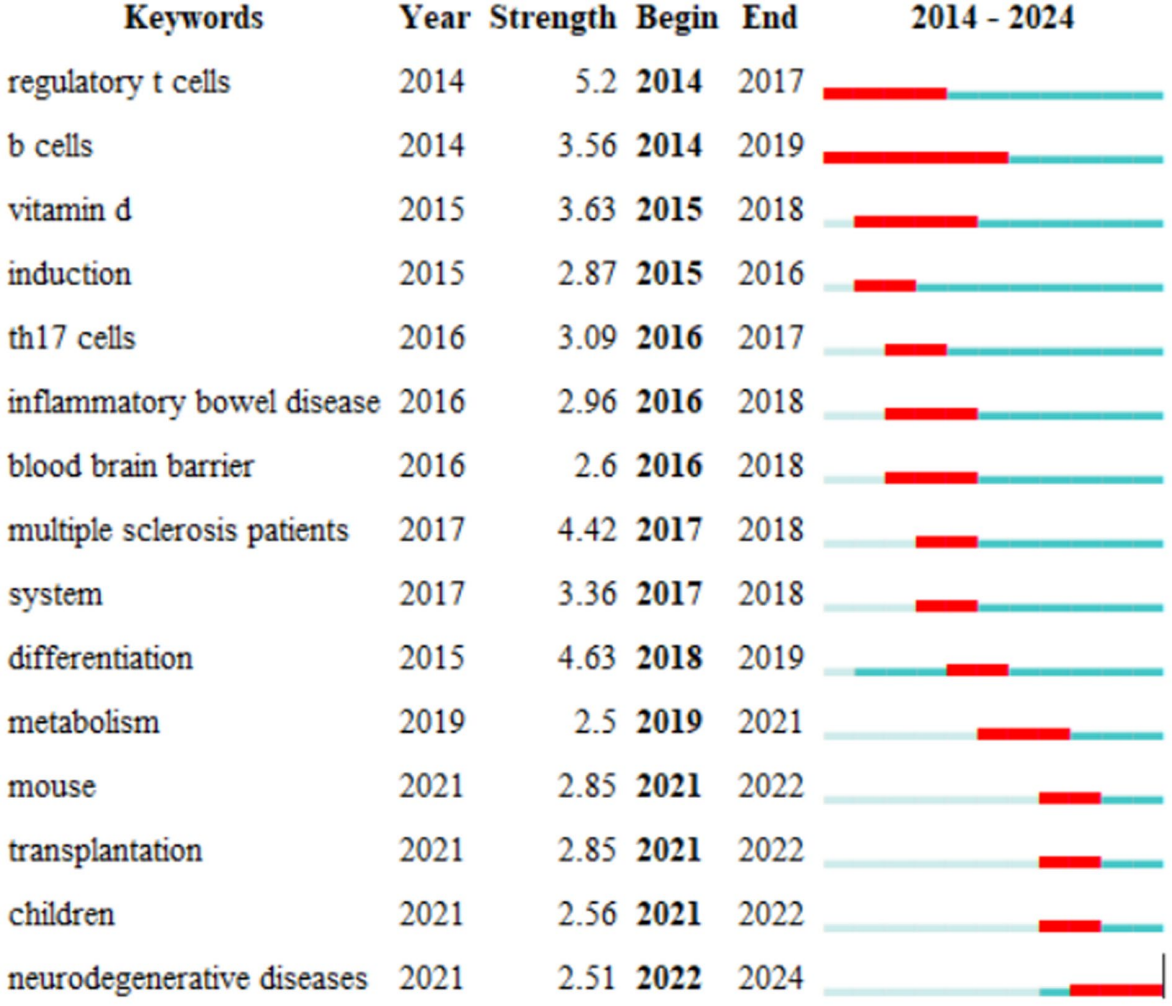
The top 15 keywords with the strongest citation bursts. The blue line represents the time interval, while the red line represents the period when the keyword bursts were found.

Finally, we identified 15 keywords with the strongest citation bursts since 2014 for the intestinal microbiota and demyelinating diseases using CiteSpace (Figure 8). The keyword with the strongest citation burst was found to be regulatory T cells, which are a type of T-cell in the immune system. The primary function of regulatory T cells is to regulate the immune response and prevent autoimmune diseases and excessive immune responses (Ferreira et al., 2019).

### 3.6 Highly cited publications

**Figure 9:**
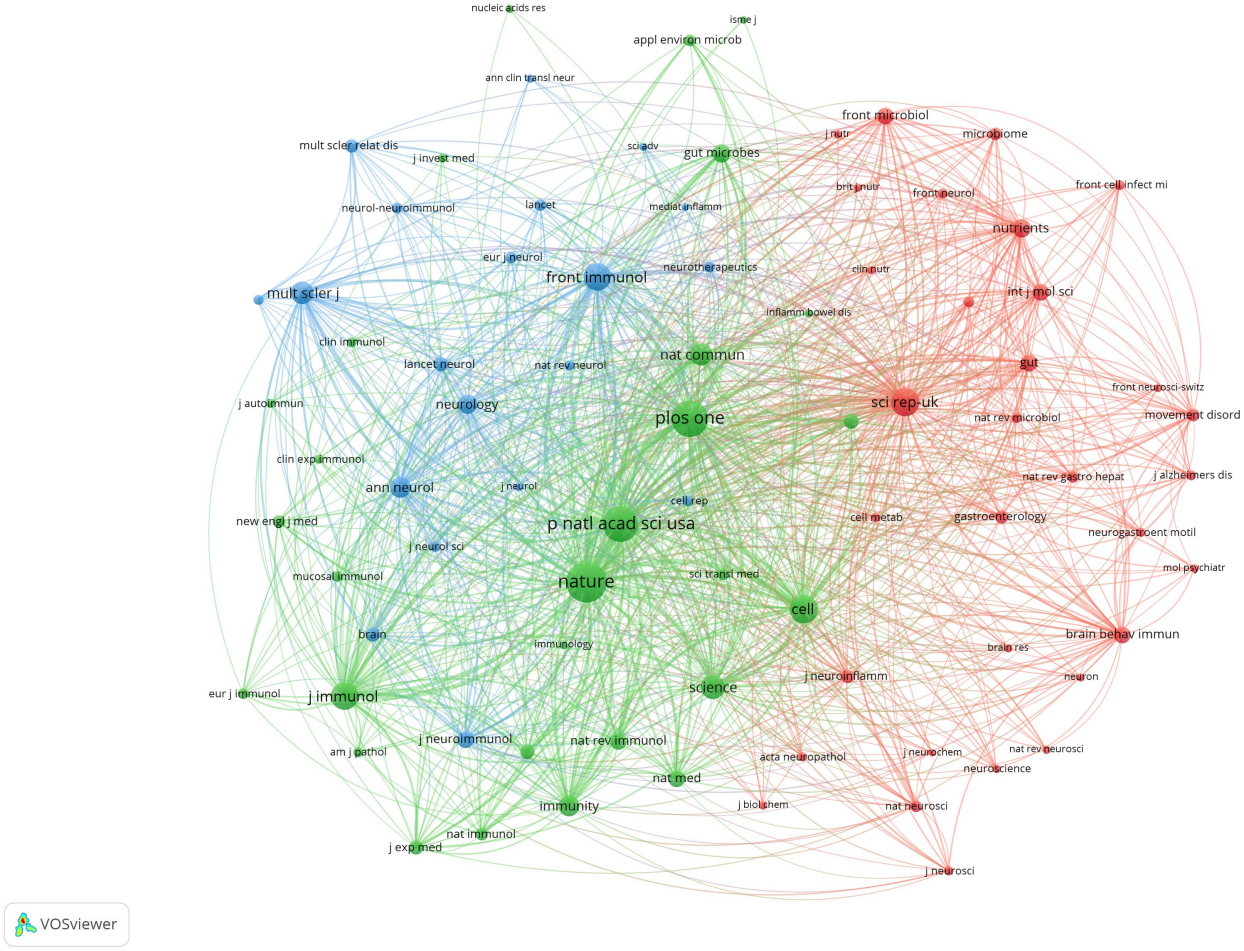
Co-Citation of cited journals.

The analysis included a total of 23,774 cited publications sourced from 3,498 journals. First, we examined the five most frequently cited publications related to the intestinal microbiota and demyelinating diseases (Table 5). The observations in Table 5 indicate that all highly cited publication types were found to be “Articles.” These publications focused on studying and analyzing the changes in and mechanisms of the gut microbiome in patients with autoimmune demyelination, including autoimmune encephalomyelitis, multiple sclerosis, and myelin oligodendrocyte glycoprotein IgG-associated disorders. However, the publication with the highest impact factor was “Commensal microbiota and myelin autoantigens cooperate to trigger autoimmune demyelination,” which was published in “Nature.” Moreover, among the top five most cited publications, three articles were published in JCR1 (JCR: Journal Citation Reports is a widely recognized tool used to evaluate the impact and influence of academic journals). Journals are often categorized into different quartiles based on their citation metrics. JCR1 refers to the top 25% of journals in a specific subject category, indicating the highest impact and most cited journals. One article is published in JCR2, which refers to the next 25% of journals, ranking between the 26th and 50th percentiles, and represents high-quality and influential journals, and One article is published in JCR3, which refers to the next 25% of journals, ranking between the 51st and 75th percentiles, and is still considered to have significant value and impact within its respective fields. The journal co-citation was observed to comprise three distinct clusters, which correspond to the three colors depicted in Figure 9. Most of the journals in the green cluster are comprehensive journals that explore the correlation between immunity and the gut microbiota. The journals in the blue cluster are dedicated to publishing articles on neurological and neuroimmune diseases. The journals in the red cluster focus on the overall study of the gut microbiota, with an emphasis on investigating the pathogenic mechanisms of the GBA in demyelinating diseases. The three journals with the highest citation frequencies were found to be Nature, PLOS One, and Proceedings of the National Academy of Sciences of the United States of America.

**Table 5:** Top five most cited publications in the research field of the intestinal microbiota and demyelinating diseases.

Rank	Title	Journal	First author	Year	Citation
1	Alterations of the human gut microbiome in multiple sclerosis	Nat Commun	Sushrut Jangi	2016	228
2	Dysbiosis in the Gut Microbiota of Patients with Multiple Sclerosis, with a Striking Depletion of Species Belonging to Clostridia XIVa and IV Clusters	PLOS One	Sachiko Miyake	2015	208
3	Multiple sclerosis patients have a distinct gut microbiota compared to healthy controls.	Sci Rep	Jun Chen	2016	191
4	Commensal microbiota and myelin autoantigen cooperate to trigger autoimmune demyelination.	Nature	Kerstin Berer	2011	172
5	Proinflammatory T-cell responses to gut microbiota promote experimental autoimmune encephalomyelitis.	Proc Natl Acad Sci U S A	Yun Kyung Lee	2011	161

## 4 Discussion

### 4.1 Status of the intestinal microbiota and demyelinating diseases

The intestines represent the most extensive microecosystem in the human body. Under optimal conditions, the balance of the gut microbiota is extremely delicate. Disruptions caused by internal or external factors can upset this equilibrium and potentially result in illness (Sochocka et al., 2019). Research has shown that the gut microbiota plays a crucial role in the regulation of brain activity, which interacts with the metabolic, peripheral immune, and central nervous systems via the GBA (Chen et al., 2021; Hamamah et al., 2022; Ullah et al., 2023). Changes in the gut microbiota have been shown to influence neurological diseases such as MS (Tremlett et al., 2017). MS, a chronic demyelinating disease with an autoimmune etiology, is a multifaceted interplay involving a variety of genetic and environmental elements and is characterized by demyelination and varying levels of damage to axons (Garg and Smith, 2015). Research has revealed that the gut microbiota, through intricate mechanisms, plays a role in the development and maturation of the central nervous system (CNS), including the process of myelination (Pröbstel et al., 2020). Therefore, we used bibliometric analysis to study the relationship between the intestinal microbiota and demyelinating diseases.

Bibliometric analyses have been conducted across various professional fields, including cardiovascular disease, infectious diseases, neurology, and oncology. However, a bibliometric analysis focusing on the intestinal microbiota and demyelinating diseases remains uncommon. Our research analyzed 429 publications from the WoSCC database on the intestinal microbiota and demyelinating diseases using VOSviewer, CiteSpace, and Charticulator. Research on the intestinal microbiota and demyelinating diseases has shown a steadily increasing trend. However, from 2014 to 2016, studies in this field received limited attention from researchers, with an average annual publication output of only 14 articles. From 2017 to the study’s deadline, there was a notable surge in the number of publications, averaging 54.1 articles per year. This trend suggests that the field of the intestinal microbiota and demyelinating diseases is currently undergoing rapid growth and garnering increasing attention from the academic community.

The USA, the People’s Republic of China, and Italy are the leading countries in research on the relationships between the intestinal microbiota and demyelinating diseases (*n* = 261, 60.8%). Among the three countries, the USA ranks first, and approximately 80% of the top five organizations are located in the USA. The USA has made the most substantial contribution to the literature in the intestinal microbiota and demyelinating diseases field, emphasizing the significant research contributions made by the USA in this field. In addition, our findings indicate that the USA has the most extensive collaborations with other countries, which is conducive to the long-term development of research on the intestinal microbiota and demyelinating diseases. Therefore, we recommend that organizations worldwide engage in broad, cross-institutional, and even cross-national collaborations to further advance the understanding of the correlation between the intestinal microbiota and demyelinating diseases. Among the top five organizations in terms of the number of publications, three are tied for fourth place and six are tied for fifth place. Half of these organizations are based in the USA, including the University of California, San Francisco, Harvard Medical School, Mayo Clinic, University of Iowa, Dartmouth College, and University of Utah. The USA has received a high level of attention and importance in the field of the intestinal microbiota and demyelinating diseases. Based on these data, to advance the field, increased collaboration between more countries and institutions in the intestinal microbiota and demyelinating disease research is necessary.

From the perspective of the author, when evaluating prolific authors, it is essential to consider both the number of publications and the number of citations they have received. In terms of the number of publications, Helen Tremlett from the University of British Columbia, Canada, has the highest number of publications, followed by Emmanuelle Waubant, Lloyd H. Kasper, Javier Ocho-reparaz, and Ashutosh K. Mangalam. Her research has focused on the etiology, risk factors, and epidemiology of MS, as well as gut microbiome research in demyelinating diseases. She and her colleagues published an article in the Lancet Neurol and conducted the first systematic exploration of risk factors for multiple sclerosis, laying the foundation for subsequent investigations into the disease’s underlying mechanisms and potential interventions (McKay and Tremlett, 2015). Furthermore, Helen Tremlett and her colleagues identified the gut microbiome commonly linked to pediatric patients with multiple sclerosis and established a sizable biobank of stool samples from children with MS, published in JAMA Neurology (Tremlett and Waubant, 2018). Her significant contribution to the intestinal microbiota and demyelinating disease field suggests that she has been actively involved in advancing the knowledge and understanding of this field. Regarding publication citations, Lloyd H. Kasper from Dartmouth College has the highest number of publication citations, with an average of 86.69 citations. Following him are Ashutosh K. Mangalam, Emmanuelle Waubant, Helen Tremlett, and Javier Ocho-reparaz. A high number of citations usually indicates that these publications have a significant influence on the academic community and are widely acknowledged and referenced. The finding reflects the contributions and reputation of Lloyd H. Kasper’s team or academic institution within the field of the intestinal microbiota and demyelinating diseases. It has had a substantial impact on the academic community as his work has been widely recognized and referenced by other researchers.

Regarding the number of publications, the top five most active journals are Frontiers in Immunology (n = 33), International Journal of Molecular Sciences (n = 21), Scientific Reports (n = 12), Multiple Sclerosis Journal (n = 14), and Multiple Sclerosis and Related Disorders (n = 13). As the journal with the highest number of publications and total citations in this field, Frontiers in Immunology (IF: 7.3 JCR2) holds a leading position in research on the intestinal microbiota and demyelinating diseases. Nevertheless, Scientific Reports (IF: 4.2 JCR2) has the highest average citations/publications. This inconsistency highlights the profound influence and quality of the publications in Frontiers in Immunology.

Most cited publications are works that have been referenced by other researchers. They play a crucial role in academic research by providing a foundation for new studies, acknowledging the contributions of previous studies, and helping to build a comprehensive understanding of a particular field. In our research, we found that the top five most cited publications are committed to studying and analyzing the changes in and mechanism of the gut microbiome in patients with autoimmune demyelination, including autoimmune encephalomyelitis, multiple sclerosis, and myelin oligodendrocyte glycoprotein IgG-associated disorders. The publication with the greatest impact factor was found to be “Commensal microbiota and myelin autoantigens cooperate to trigger autoimmune demyelination,” which was published in “Nature.” The study presented the first description of the sequential roles of intact gut microbiota and myelin autoantigens in the onset of a sophisticated, spontaneous demyelinating autoimmune condition. Furthermore, the authors suggested that dietary risk factors may play a role, potentially contributing to the noticeable rise in the incidence of multiple sclerosis in Asian countries, such as Japan, a phenomenon often attributed to the adoption of a “Westernized” lifestyle (Berer et al., 2011).

### 4.2 Hot topics and trends in intestinal microbiota and demyelinating disease research

Keywords represent the words of the core and are highly refined in an article. In bibliometrics frequently used keywords help pinpoint the main areas of focus and emerging trends within a specific research domain. Based on the cluster analysis and keyword co-occurrence map produced using CiteSpace and VOSviewer it was found that the majority of scholars focused on “multiple sclerosis (n = 284)” and “gut microbiota (n = 166)” in the field of the intestinal microbiota and demyelinating diseases which led to the identification of keywords. In demyelinating diseases of the nervous system the relationships between MS and the gut microbiota have been extensively studied (Correale et al., 2022; Altieri et al., 2023; Wang et al., 2022). Currently the primary research focus is on the GBA MS neurological disorders microbial diversity the ketogenic diet and bacillus

The GBA acts as a bidirectional link between the gut microbiome and the immune system, potentially enhancing our understanding of the pathophysiology of inflammatory demyelinating diseases (Camara-Lemarroy et al., 2018). The communication between the vagus nerve and enteric nervous system is an important connection between the CNS and the gut. One of the pathways through which the brain communicates with the intestinal microbiota involves direct interaction via the vagus nerve with the spinal cord (Shokufeh Ghasemian et al., 2022). Gut microbiota establishes a direct neural link between the brain and gastrointestinal microbiota via the vagus nerve and the activation of the enteric nervous system afferent neurons (Faraji et al., 2025). In addition, they can transmit microbial signals either through the diffusion of compounds or with the help of other cells (Bonaz et al., 2018), highlighting the complicated interplay and pathophysiology between the gut and CNS. Another crucial link between the CNS and the gut is through the bloodstream. The gut microbiome can generate a wide array of microbial components, metabolites, and neurotransmitters, which are released into the bloodstream and directly influence the CNS to regulate the host’s neural function and immune response (Lai et al., 2021). In other words, communication between the brain and the gastrointestinal tract is bidirectional and intricate.

MS is an autoimmune disease of the CNS characterized by demyelination, axonal damage, and degeneration, and increasing evidence indicates that the GBA may play a vital role in neurological disorders such as MS (iMSMS Consortium, 2022; Parodi et al., 2021). A previous study revealed that changes in the composition of the intestinal microbiota, leading to an impaired immune system, could initiate the development of MS or exacerbate its symptoms (Fan and Zhang, 2019). Diet and dietary elements significantly influence the composition of the gut microbiota and are key factors in altering the bacterial flora. A ketogenic diet can alter the composition of the gut microbiota (Beam et al., 2021; Olson et al., 2018). MS causes damage to the myelin sheaths that protect neurons, thereby disrupting the normal transmission of nerve impulses. Research indicates that a ketogenic diet may contribute to the potential reconstruction and repair of the myelin sheaths (Dyńka et al., 2022). Objective evidence suggests that the use of a ketogenic diet in the treatment of MS is recognized. It can influence the progression and prevention of the disease while also being safe and feasible to implement (Lin et al., 2022).

Moreover, Berer et al. demonstrated that gut commensal bacteria could initiate a relapsing–remitting autoimmune disease characterized by demyelination and driven by myelin-specific CD4+ T cells in transgenic mice (Berer et al., 2011). In addition, among peripheral immune cells, intestinal Th17 cells have been reported to play a crucial role in the immunopathology of MS (Steinman, 2001). Research has revealed that Th17 cells are prevalent in the peripheral blood, cerebrospinal fluid, and brain lesions of individuals with MS, with elevated levels and increased levels of inflammatory mediators observed during disease recurrence (Moser et al., 2020). These findings indicate that reducing pathogenic Th17 cells may help prevent the recurrence of autoimmunity in individuals with MS (Sie et al., 2014; Issazadeh-Navikas et al., 2012). Similarly, alterations in the gut microbiota or its metabolites can also modulate inflammation and demyelination in experimental autoimmune encephalomyelitis (Lee et al., 2011). Evidence indicates that changes in the composition and diversity of the microbiome may affect the progression of MS (Zangeneh et al., 2021). In the treatment of relapsing–remitting MS, monoclonal B-cell-depleting antibodies have shown significant efficacy in controlling inflammation (Laurent et al., 2023). Researchers analyzed oral swab samples using 16S rDNA sequencing followed by bioinformatic analyses. The study revealed that patients with MS displayed lower alpha diversity in their oral microbiota patterns, while an increase in alpha diversity was observed in the gut microbiota of these patients following B-cell depletion (Troci et al., 2022). The relationship between changes in the intestinal microbiota and MS has not been thoroughly studied and necessitates further investigation.

CD4+ T cells (Th17 cells), which produce IL-17, are implicated in the development of various autoimmune and inflammatory diseases and are known to play a significant role in the intestinal mucosa (Schnell et al., 2023). Research has confirmed that human Th17 lymphocytes contribute to the disruption of the blood–brain barrier and that antigen-specific Th17 cells migrate through the choroid plexus into the subarachnoid space and then become reactivated (Kebir et al., 2007). A significant influx of activated Th17 cells and other inflammatory cells into the white matter of the CNS leads to tissue damage, including demyelination, ultimately causing neurological deficits (Zepp et al., 2011). During acute episodes of MS, particularly in patients with relapsing–remitting MS, there is a notable increase in the frequency of Th17 cells in both the peripheral blood and cerebrospinal fluid (Matusevicius et al., 1999). Moreover, successful therapeutic interventions in patients with MS have been linked to a decrease in Th17 cells in the peripheral blood (Mehling et al., 2010; Durelli et al., 2009). Chris et al. confirmed that the concentration of IL-17 in patients with MS is directly proportional to the number of active lesions observed through magnetic resonance imaging (MRI) (Hedegaard et al., 2008).

Furthermore, a connection has been established between Th17 cells and the pathogenesis of neuromyelitis optica (NMO), along with its spectrum of disorders known as NMOSD. In patients with NMO who test positive for AQP4-IgG, a defining characteristic is astrocyte damage that results in secondary demyelination (Dos Passos et al., 2016). In conclusion, Th17 cells might be important in driving the pathology of demyelinating diseases.

### 4.3 Strengths and limitations

Our study provides the first comprehensive systematic bibliometric analysis of the intestinal microbiota and demyelinating diseases, offering extensive insights and directions for both clinical practitioners and academic researchers in this domain. The application of bibliometric techniques, coupled with visual methods, enables scientists to gain a clear and direct understanding of focal areas, progression, and emerging trends within research on the intestinal microbiota and demyelinating conditions. However, our study also has limitations. Our analysis was limited to articles that appeared on WoSCC during a designated period and were written in English, which might have led to the exclusion of findings from alternative databases. Nevertheless, using visual methods to discern the prevailing conditions, focal points, and directional shifts within an academic domain continues to be beneficial.

## 5 Conclusion

Using VOSviewer, CiteSpace, and Charticulator, we conducted a bibliometric analysis of articles related to the intestinal microbiota and demyelinating diseases. Following the identification of significant articles, authors, journals, institutions, and countries, we conducted an additional analysis of the research network. Owing to the increasing annual publication volume of related literature, the relationship between the intestinal microbiota and demyelinating diseases has attracted growing attention. Regarding the intestinal microbiota and demyelinating diseases, Helen Tremlett stands out as the most prolific author, having published 30 articles that have garnered 659 citations. The USA, the People’s Republic of China, and Italy are the leading countries where the relationships between the intestinal microbiota and demyelinating diseases have been studied. Furthermore, while there is currently limited collaboration among various countries, strengthening partnerships and communication between different nations and organizations is crucial. The publications of Helen Tremlett and Lloyd H. Kasper have shaped the current understanding of the role of the gut microbiota in demyelinating diseases. Furthermore, this comprehensive analysis underscores the importance of continued research into the role of the gut microbiome in disease, particularly in the context of demyelinating diseases such as multiple sclerosis. The relationships and underlying processes linking the gut microbiota to demyelinating diseases remain key research topics. The insights gained from this analysis will help guide future research directions, potentially leading to breakthroughs in the prevention, diagnosis, and treatment of these complex conditions.

## Data Availability

The original contributions presented in the study are included in the article/supplementary material, further inquiries can be directed to the corresponding author.
