# Correction: Bibliometric analysis of the intestinal microbiota and demyelinating diseases, particularly multiple sclerosis, since 2014

**DOI:** 10.3389/fnins.2025.1635017

**Published:** 2025-06-10

**Authors:** Ling Chen, Le-Le Wu, Chang-Yin Yu, Zu-Cai Xu, Hao Huang

**Affiliations:** ^1^Department of Neurology, Affiliated Hospital of Zunyi Medical University, Zunyi, China; ^2^Department of Neurology, Xinqiao Hospital and the Second Affiliated Hospital, Army Medical University (Third Military Medical University), Chongqing, China

**Keywords:** intestinal microbiota, multiple sclerosis, demyelinating diseases, bibliometrics, visualization analysis

In the published article, the reference for (Neil, 2017) was incorrectly written as Neil, J. V. (2017). Animal communication: when I'm calling you, will you answer too? *Curr. Biol*. 27, R713–R715. doi: 10.1016/j.cub.2017.05.064. It should be (Faraji, 2025) and the full reference is Faraji, N., Payami, B., Ebadpour, N., and Gorji, A. (2025). Vagus nerve stimulation and gut microbiota interactions: a novel therapeutic avenue for neuropsychiatric disorders. *Neurosci. Biobehav. Rev*. 169:105990. doi: 10.1016/j.neubiorev.2024.105990.

The authors apologize for this error and state that this does not change the scientific conclusions of the article in any way. The original version of this article has been updated.

